# 1,6-Bis(chloro­meth­yl)pyridine

**DOI:** 10.1107/S1600536811020678

**Published:** 2011-06-04

**Authors:** Richard Betz, Thomas Gerber, Henk Schalekamp

**Affiliations:** a Nelson Mandela Metropolitan University, Summerstrand Campus, Department of Chemistry, University Way, Summerstrand, PO Box 77000, Port Elizabeth 6031, South Africa

## Abstract

In the title compound, C_7_H_7_Cl_2_N, a halogenated derivative of 2,6-lutidine, the C—Cl vectors of the chloro­methyl groups point at opposite sides of the aromatic plane to each other. A weak dispersive Cl⋯Cl contact [3.4342 (3) Å] connects the mol­ecules into a chain along the [101] direction. A π–π inter­action with a centroid–centroid distance of 3.7481 (5) Å is also observed.

## Related literature

For the crystal structure of the hydro­chloride of the title compound, see: Lozano & Jones (2004 ▶).
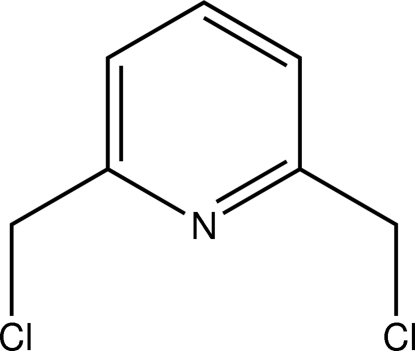


## Experimental

### 

#### Crystal data

C_7_H_7_Cl_2_N
*M* *_r_* = 176.04
Monoclinic, 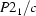

*a* = 8.9927 (2) Å
*b* = 12.1581 (3) Å
*c* = 7.4893 (2) Å
β = 113.535 (1)°
*V* = 750.72 (3) Å^3^
*Z* = 4
Mo *K*α radiation
μ = 0.78 mm^−1^
*T* = 100 K
0.52 × 0.41 × 0.18 mm

#### Data collection

Bruker APEXII CCD diffractometer
Absorption correction: multi-scan (*SADABS*; Bruker, 2008 ▶) *T*_min_ = 0.727, *T*_max_ = 0.872
6994 measured reflections
1845 independent reflections
1768 reflections with *I* > 2σ(*I*)
*R*_int_ = 0.014

#### Refinement

*R*[*F* ^2^ > 2σ(*F* ^2^)] = 0.019
*wR*(*F* ^2^) = 0.051
*S* = 1.09
1845 reflections
91 parameters
H-atom parameters constrained
Δρ_max_ = 0.36 e Å^−3^
Δρ_min_ = −0.18 e Å^−3^

### 

Data collection: *APEX2* (Bruker, 2010 ▶); cell refinement: *SAINT* (Bruker, 2010 ▶); data reduction: *SAINT*; program(s) used to solve structure: *SHELXS97* (Sheldrick, 2008 ▶); program(s) used to refine structure: *SHELXL97* (Sheldrick, 2008 ▶); molecular graphics: *ORTEP-3* (Farrugia, 1997 ▶) and *Mercury* (Macrae *et al.*, 2008 ▶); software used to prepare material for publication: *SHELXL97* and *PLATON* (Spek, 2009 ▶).

## Supplementary Material

Crystal structure: contains datablock(s) I, global. DOI: 10.1107/S1600536811020678/is2723sup1.cif

Supplementary material file. DOI: 10.1107/S1600536811020678/is2723Isup2.cdx

Structure factors: contains datablock(s) I. DOI: 10.1107/S1600536811020678/is2723Isup3.hkl

Supplementary material file. DOI: 10.1107/S1600536811020678/is2723Isup4.cml

Additional supplementary materials:  crystallographic information; 3D view; checkCIF report

## Figures and Tables

**Fig. 1.**
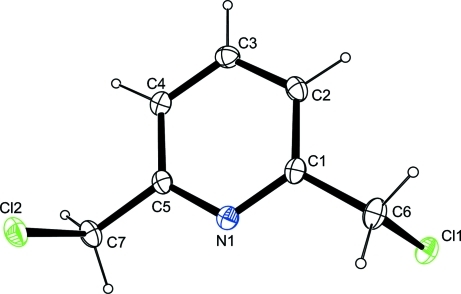
The molecular structure of the title compound, with atom labels and anisotropic displacement ellipsoids (drawn at 50% probability level).

**Fig. 2.**
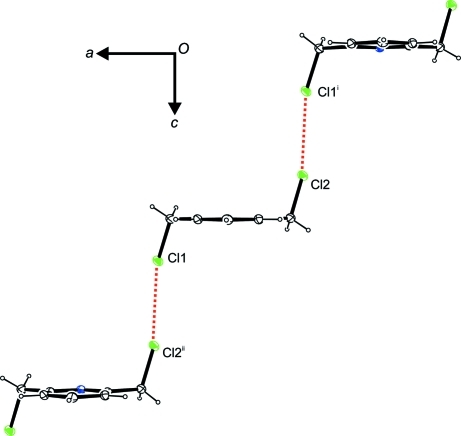
Intermolecular contacts, viewed along the b axis. Symmetry codes: (i) x + 1, y, z + 1; (ii) x - 1, y, z - 1.

**Fig. 3.**
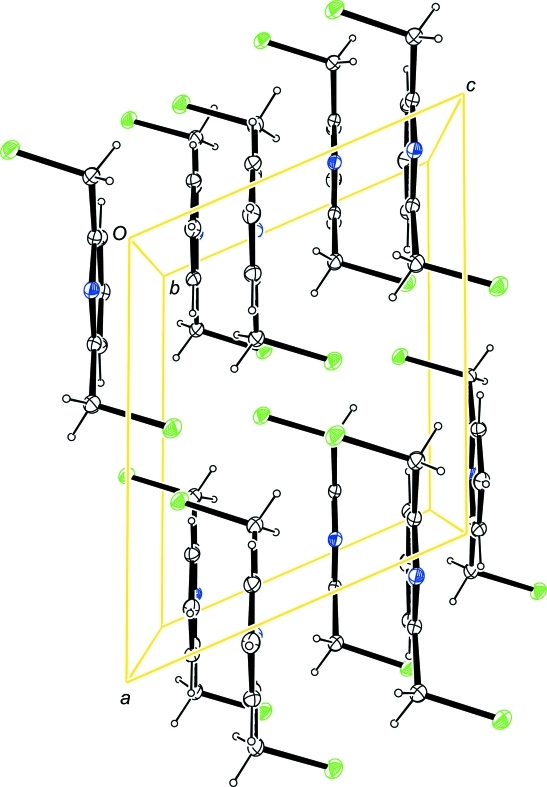
Molecular packing of the title compound, viewed along the b axis. (anisotropic displacement ellipsoids drawn at 50% probability level).

## supplementary crystallographic information

### Comment

Multidentate ligands are versatile complexation agents for a variety of main group elements as well as transition metals. In some cases, the assignment or the determination of oxidation states of coordinated transition metals relies heavily on the knowledge of metric parameters within the ligand. To enable comparative studies within a group of coordination compounds currently under investigation in our working group we determined the crystal structure of the title compound which serves as a starting material for one of the multidentate ligands applied thereof. The crystal structure of the hydrochloride of the title compound is apparent in the literature (Lozano & Jones, 2004).

In the molecule, the chloromethyl substituents are pointing at the two different sides of the plane defined by the atoms of the aromatic system. Intracyclic angles span a range from 117.41 (8)–123.38 (8) ° with the smallest angle on the nitrogen atom and the two biggest ones on the carbon atoms adjacent to it (Fig. 1).

The molecules in the crystal structure only interact *via* weak dispersive Cl···Cl contacts [Cl1···Cl2^ii^ 3.4342 (3) Å; symmetry code: (ii) *x* - 1, *y*, *z* - 1] whose range falls by about 0.1 Å below the sum of van-der-Waals radii. These contacts connect the molecules to a chains along the [101] direction (Fig. 2). The closest distance between two π-systems was found at 3.7481 (5) Å. The packing of the compound in the crystal structure is shown in Figure 3.

### Experimental

The compound was obtained commercially (Aldrich). Crystals suitable for the X-ray diffraction study were taken directly from the provided product.

### Refinement

C-bound H atoms were placed in calculated positions (C—H 0.95 Å for aromatic carbon atoms and C—H 0.99 Å for the methylene groups) and were included in the refinement in the riding model approximation, with *U*_iso_(H) set to 1.2*U*_eq_(C).

### Crystal data

**Table:**

C_7_H_7_Cl_2_N	*F*(000) = 360
*M**_r_* = 176.04	*D*_x_ = 1.558 Mg m^−^^3^
Monoclinic, *P*2_1_/*c*	Melting point: 346 K
Hall symbol: -P 2ybc	Mo *K*α radiation, λ = 0.71073 Å
*a* = 8.9927 (2) Å	Cell parameters from 5840 reflections
*b* = 12.1581 (3) Å	θ = 2.5–28.3°
*c* = 7.4893 (2) Å	µ = 0.78 mm^−^^1^
β = 113.535 (1)°	*T* = 100 K
*V* = 750.72 (3) Å^3^	Platelet, colourless
*Z* = 4	0.52 × 0.41 × 0.18 mm

### Data collection

**Table:**

Bruker APEXII CCD diffractometer	1845 independent reflections
Radiation source: fine-focus sealed tube	1768 reflections with *I* > 2σ(*I*)
graphite	*R*_int_ = 0.014
φ and ω scans	θ_max_ = 28.3°, θ_min_ = 2.5°
Absorption correction: multi-scan (*SADABS*; Bruker, 2008)	*h* = −11→11
*T*_min_ = 0.727, *T*_max_ = 0.872	*k* = −14→16
6994 measured reflections	*l* = −9→9

### Refinement

**Table:**

Refinement on *F*^2^	Primary atom site location: structure-invariant direct methods
Least-squares matrix: full	Secondary atom site location: difference Fourier map
*R*[*F*^2^ > 2σ(*F*^2^)] = 0.019	Hydrogen site location: inferred from neighbouring sites
*wR*(*F*^2^) = 0.051	H-atom parameters constrained
*S* = 1.09	*w* = 1/[σ^2^(*F*_o_^2^) + (0.0223*P*)^2^ + 0.2778*P*] where *P* = (*F*_o_^2^ + 2*F*_c_^2^)/3
1845 reflections	(Δ/σ)_max_ = 0.001
91 parameters	Δρ_max_ = 0.36 e Å^−^^3^
0 restraints	Δρ_min_ = −0.18 e Å^−^^3^

### Fractional atomic coordinates and isotropic or equivalent isotropic displacement parameters (Å^2^)

**Table:**

	*x*	*y*	*z*	*U*_iso_*/*U*_eq_	
Cl1	−0.34591 (3)	0.401927 (19)	0.11753 (3)	0.01804 (7)	
Cl2	0.47219 (3)	0.37996 (2)	0.61934 (3)	0.01866 (7)	
N1	0.06320 (9)	0.35805 (7)	0.36585 (10)	0.01355 (16)	
C1	−0.06797 (11)	0.30473 (8)	0.36501 (12)	0.01361 (17)	
C2	−0.07993 (11)	0.19055 (8)	0.36280 (13)	0.01536 (18)	
H2	−0.1747	0.1559	0.3626	0.018*	
C3	0.04877 (11)	0.12810 (8)	0.36096 (13)	0.01600 (18)	
H3	0.0435	0.0500	0.3585	0.019*	
C4	0.18529 (11)	0.18224 (8)	0.36276 (13)	0.01472 (18)	
H4	0.2757	0.1420	0.3624	0.018*	
C5	0.18709 (10)	0.29671 (8)	0.36515 (12)	0.01296 (17)	
C6	−0.20508 (12)	0.37527 (8)	0.36486 (13)	0.01779 (19)	
H6A	−0.1617	0.4457	0.4317	0.021*	
H6B	−0.2620	0.3375	0.4365	0.021*	
C7	0.33299 (11)	0.35840 (8)	0.36959 (13)	0.01679 (19)	
H7A	0.2991	0.4302	0.3033	0.020*	
H7B	0.3872	0.3160	0.2998	0.020*	

### Atomic displacement parameters (Å^2^)

**Table:**

	*U*^11^	*U*^22^	*U*^33^	*U*^12^	*U*^13^	*U*^23^
Cl1	0.01397 (11)	0.02042 (12)	0.01663 (12)	0.00406 (8)	0.00284 (8)	0.00130 (8)
Cl2	0.01427 (11)	0.02260 (13)	0.01632 (12)	−0.00528 (8)	0.00316 (8)	−0.00105 (8)
N1	0.0133 (4)	0.0151 (4)	0.0111 (3)	0.0002 (3)	0.0037 (3)	−0.0007 (3)
C1	0.0118 (4)	0.0183 (4)	0.0093 (4)	0.0014 (3)	0.0027 (3)	−0.0005 (3)
C2	0.0126 (4)	0.0193 (4)	0.0131 (4)	−0.0030 (3)	0.0040 (3)	−0.0002 (3)
C3	0.0177 (4)	0.0136 (4)	0.0153 (4)	−0.0009 (3)	0.0050 (3)	−0.0006 (3)
C4	0.0134 (4)	0.0165 (4)	0.0137 (4)	0.0016 (3)	0.0048 (3)	−0.0008 (3)
C5	0.0118 (4)	0.0165 (4)	0.0093 (4)	−0.0014 (3)	0.0029 (3)	−0.0008 (3)
C6	0.0143 (4)	0.0243 (5)	0.0135 (4)	0.0042 (4)	0.0042 (3)	−0.0007 (3)
C7	0.0146 (4)	0.0218 (5)	0.0134 (4)	−0.0046 (3)	0.0049 (3)	−0.0021 (3)

### Geometric parameters (Å, °)

**Table:**

Cl1—C6	1.8074 (10)	C3—H3	0.9500
Cl2—C7	1.8068 (9)	C4—C5	1.3918 (13)
N1—C5	1.3425 (11)	C4—H4	0.9500
N1—C1	1.3438 (12)	C5—C7	1.5001 (12)
C1—C2	1.3920 (14)	C6—H6A	0.9900
C1—C6	1.5016 (13)	C6—H6B	0.9900
C2—C3	1.3888 (13)	C7—H7A	0.9900
C2—H2	0.9500	C7—H7B	0.9900
C3—C4	1.3883 (13)		
			
C5—N1—C1	117.41 (8)	N1—C5—C7	116.23 (8)
N1—C1—C2	123.00 (8)	C4—C5—C7	120.39 (8)
N1—C1—C6	116.32 (8)	C1—C6—Cl1	110.02 (6)
C2—C1—C6	120.67 (8)	C1—C6—H6A	109.7
C3—C2—C1	118.98 (9)	Cl1—C6—H6A	109.7
C3—C2—H2	120.5	C1—C6—H6B	109.7
C1—C2—H2	120.5	Cl1—C6—H6B	109.7
C4—C3—C2	118.55 (9)	H6A—C6—H6B	108.2
C4—C3—H3	120.7	C5—C7—Cl2	109.50 (6)
C2—C3—H3	120.7	C5—C7—H7A	109.8
C3—C4—C5	118.68 (8)	Cl2—C7—H7A	109.8
C3—C4—H4	120.7	C5—C7—H7B	109.8
C5—C4—H4	120.7	Cl2—C7—H7B	109.8
N1—C5—C4	123.38 (8)	H7A—C7—H7B	108.2
			
C5—N1—C1—C2	−0.23 (12)	C1—N1—C5—C7	−178.98 (7)
C5—N1—C1—C6	−179.72 (7)	C3—C4—C5—N1	0.01 (13)
N1—C1—C2—C3	−0.14 (14)	C3—C4—C5—C7	179.26 (8)
C6—C1—C2—C3	179.33 (8)	N1—C1—C6—Cl1	91.43 (8)
C1—C2—C3—C4	0.44 (13)	C2—C1—C6—Cl1	−88.07 (9)
C2—C3—C4—C5	−0.38 (13)	N1—C5—C7—Cl2	90.05 (8)
C1—N1—C5—C4	0.29 (12)	C4—C5—C7—Cl2	−89.24 (9)
